# Cannabis Use Disorders Lead to Hospitalizations for Peptic Ulcer Disease: Insights From a Nationwide Inpatient Sample Analysis

**DOI:** 10.7759/cureus.15405

**Published:** 2021-06-02

**Authors:** Hajara Joundi, Kristal N Pereira, Goher Haneef, Renu Bhandari, Jannat Malik, Rushi P Shah, Albulena Sejdiu, Keerthika Mathialagan

**Affiliations:** 1 Internal Medicine, Cadi Ayyad University, Marrakesh, MAR; 2 Internal Medicine, Terna Medical College, Mumbai, IND; 3 Internal Medicine, University of Health Sciences, Lahore, PAK; 4 Emergency Medicine, University of Cincinnati Medical Center, Cincinnati, USA; 5 Medicine, Manipal College of Medical Sciences, Kaski, NPL; 6 Family Medicine, National University of Medical Sciences, Rawalpindi, PAK; 7 Medicine, Byramjee Jeejeebhoy Medical College, Rajkot, IND; 8 Psychiatry, Cyril and Methodius University, Kumanovo, MKD; 9 Psychiatry, Sree Balaji Medical College and Hospital, Chennai, IND

**Keywords:** peptic ulcer, cannabis use disorder, cannabis abuse, substance recreational use, risk factors

## Abstract

Objectives

In this study, we aimed to explore the independent association between cannabis use disorders (CUD) and peptic ulcer disease (PUD)-related hospitalization, and then to delineate the demographic differences among PUD inpatients with versus without CUD.

Methodology

We conducted a cross-sectional study using the Nationwide Inpatient Sample of 50,444,133 patients. We then subgrouped them into PUD and non-PUD cohorts. We compared non-PUD and PUD cohorts using bivariate analysis to delineate the differences in demographics and comorbid risk factors (chronic lung disease, chronic kidney disease, liver disease, diabetes, chronic nonsteroidal anti-inflammatory drug use, tobacco abuse, and alcohol abuse). We used logistic regression analysis to measure the odds ratio (OR) of the association between CUD and PUD-related hospitalization.

Results

The prevalence of PUD was 0.14% (N = 70,898) among the total inpatient population. It was more prevalent in whites (65%) and males were at higher odds (OR: 1.11; P < 0.001) of being hospitalized for PUD. After controlling for potential comorbid risk factors and demographic confounders, the odds of association between CUD and PUD-related hospitalization were statistically significant (OR: 1.18; P < 0.001).

Conclusions

CUD was associated with a modest but significant increase of 18% in the likelihood of hospitalization for PUD. With the legalization of cannabis use and its increasing and problematic consumption, it is imperative to understand the impact of cannabis use on the physical health of patients and the related gastrointestinal problems.

## Introduction

Peptic ulcer disease (PUD) affects about 4.6 million people, with an incidence of 0.1-0.3% per year in the United States [1]. PUD affects both men and women equally, with a mortality rate of one death per 10,000 cases [2]. PUD posed a significant threat in the past because of the higher morbidity and mortality associated with the disease. Two major risk factors for PUD are *Helicobacter pylori* infection and nonsteroidal anti-inflammatory drug (NSAID) use [3]. Other risk factors include genetic predisposition, lifestyle practices, stress mismanagement, and low socioeconomic status [4]. Even though the incidence of *H. pylori*-associated PUD has declined, NSAID-induced PUD is still a significant concern.

Recently, the trend of cannabis use has been increasing in the United States. It is the most commonly used illicit drug, particularly in adults [5]. According to the National Institute of Drug Abuse survey, the lifetime prevalence of cannabis abuse and/ dependence among adults aged 26 or older was 47.8% in 2018 [6]. Cannabinoids, both natural and synthetic forms, have become the most accessible and used illicit drugs by adolescents and adults. As a result of their potential for addiction and harmful side effects, the legalization of cannabinoids and the use of their by-products in the medical field have long been controversial [7]. But since the legalization of cannabis for medical conditions, there has been a rising trend in its unconventional use.

Cannabinoid receptor agonists are effective against inflammatory and neuropathic pain refractory to standard therapy [8]. Cannabinoids work on the gastrointestinal (GI) tract through the cannabinoid cb1 receptors in the myenteric and submucosal nerve plexuses. It reduces gastric motility and acid secretion and relaxes the lower esophageal sphincter. Thus, it can provide gastroprotective benefits, potentially helping heal gastric ulcers [9,10]. Additional exploration of this system could offer new therapeutic alternatives in GI diseases such as gastric ulcers and gastroesophageal reflux disease [11]. While cannabis could be gastroprotective, studies also highlight the possible negative GI outcomes, but there exists limited literature on this important area. Cannabinoid hyperemesis syndrome, pancreatitis, and hepatotoxicity are some of the negative outcomes of recreational cannabis use [7].

Hence, the risks associated with cannabis use outweigh the benefits. Apart from the risk of abuse potential, both cannabis use and cannabis use disorders (CUD) put an individual at risk of developing psychiatric comorbidities such as depression and anxiety [12]. Ultimately, cannabis use will always represent a threat to individual quality of life, overall functioning, and wellbeing. Thus, we conducted a cross-sectional study to explore the independent association of CUD and odds for hospitalizations for PUD. Next, we delineated the demographic differences among PUD inpatients with versus without CUD.

## Materials and methods

We conducted a cross-sectional study using the Nationwide Inpatient Sample (NIS, 2010 to 2014). The NIS is the largest inpatient database covering about 4,400 hospitals across 44 states in the United States. As the NIS is the largest publicly available de-identified database with significant protection of the patient identity, this study did not require approval from an institutional review board [13].

We included 50,444,133 patients (age: 18-50) with a primary discharge diagnosis of medical illnesses and further grouped by a primary diagnosis of PUD (N = 70,898). We included demographic characteristics (age, gender, and race/ethnicities) and comorbid risk factors for PUD based on the current literature (chronic lung disease, chronic kidney disease, liver disease, diabetes, chronic NSAID use, tobacco abuse, alcohol abuse, and CUD) [14].

We compared non-PUD and PUD cohorts using bivariate analysis to delineate the differences in demographics and comorbid risk factors. Then, logistic regression analysis was conducted to determine the odds ratio (OR) of association between CUD and PUD-related hospitalization and then adjusting the model for demographics and comorbid risk factors. Next, we conducted a bivariate analysis to compare demographics in PUD patients with versus without CUD. All analyses were conducted using the Statistical Package for the Social Sciences (SPSS) version 26.0 (IBM Corp., Armonk, NY) with a statistical significance set a priori at a P-value of <0.05.

## Results

Our study population included 50,444,133 adult medical inpatients, of whom the majority were females (72.1%) and whites (56.2%). The prevalence of PUD was 0.14% (N = 70,898) among the total patients hospitalized in medical inpatient units. PUD was prevalent in whites (65%), followed by blacks (19.1%), and Hispanics (10.3%). Males were at higher odds (OR: 1.11; 95% confidence interval [CI]: 1.096-1.133; P < 0.001) of being hospitalized for PUD compared to females.

There existed a statistically significant association between certain risk factors and hospitalization for PUD. Patients with tobacco abuse were at higher odds of being hospitalized for PUD (OR: 2.48; 95% CI: 2.435-2.520; P < 0.001), followed by those with comorbid liver diseases (OR: 1.41; 95% CI: 1.355-1.457; P < 0.001) and alcohol abuse (OR: 1.09; 95% CI: 1.063-1.127, P < 0.001). Comorbid chronic lung diseases, chronic kidney disease, and diabetes had a negative association with PUD-related hospitalization. The odds of association between CUD and PUD-related hospitalization remained high and statistically significant (OR: 1.18; 95% CI: 1.129-1.234; P = <0.001) after controlling for potential demographic confounders and comorbid risk factors, as mentioned in Table 1.

**Table 1:** Odds of association of peptic ulcer disease hospitalization. SD: standard deviation; NSAID: nonsteroidal anti-inflammatory drugs

Variables	Peptic ulcer disease		Logistic regression model		
	(No) in %	(Yes) in %	Odds ratio	95% Confidence interval	P-value
Total patients	50,373,235	70,898	-	-	-
Mean age (SD), in years	34.0 (9.3)	39.1 (8.3)	1.05	1.050–1.052	<0.001
Gender					
Male	27.9	40.1	1.11	1.096–1.133	<0.001
Female	72.1	59.9	Reference		
Race					
White	56.2	65.0	Reference		
Black	19.0	19.1	0.97	0.953–0.992	0.007
Hispanic	16.7	10.3	0.73	0.711–0.749	<0.001
Other	8.1	5.5	0.76	0.730–0.781	<0.001
Risk factors					
No risk factor	-	-	Reference		
Chronic lung disease	9.3	12.4	0.94	0.919–0.964	<0.001
Chronic kidney disease	4.3	2.7	0.47	0.443–0.487	<0.001
Liver disease	2.3	4.9	1.41	1.355–1.457	<0.001
Diabetes	7.4	8.0	0.72	0.697–0.738	<0.001
Chronic NSAID use	0	0	-	-	-
Tobacco abuse	14.8	37.1	2.48	2.435–2.520	<0.001
Alcohol abuse	4.1	8.8	1.09	1.063–1.127	<0.001
Cannabis use disorders	1.9	3.2	1.18	1.129–1.234	<0.001

Among the PUD inpatients, 3.2% (N = 25,478) had comorbid CUD. A higher proportion of these inpatients with CUD were males. When compared with the non-CUD cohort, PUD inpatients with CUD were significantly higher in males (67.4% vs. 39.2%), blacks (36.8% vs. 18.5%), and Hispanics (13% vs. 10.2%), as shown in Table 2.

**Table 2:** Demographic characteristics of peptic ulcer disease inpatients by cannabis use disorders. SD: standard deviation

Variables	Cannabis use disorders		P-value
	(No) in %	(Yes) in %	
Total patients	640,770	25,478	-
Mean age (SD), in years	39.1 (8.3)	36.8 (8.4)	<0.001
Gender			
Male	39.2	67.4	<0.001
Female	60.8	32.6	
Race			
White	65.7	46.0	<0.001
Black	18.5	36.8	
Hispanic	10.2	13.0	
Other	5.6	4.2	

## Discussion

Although PUD is a common condition in the general population, the rates of hospitalizations have significantly decreased in the past 20 years due to a better understanding and improved quality of treatment [1]. Concordantly, only 0.14% of our study inpatient population had PUD. Our study also revealed that males were at a higher risk of PUD-related hospitalization, but previous studies have highlighted an almost equal distribution among the genders [15]. This discrepancy could be due to the lack of differentiation of the type and location of ulcers in our study as duodenal ulcers tend to be higher in males and gastric ulcers are more prevalent among females [16].

Our study found PUD to be more prevalent among whites, which coincides with results from past studies [17]. Whites have a higher incidence of idiopathic PUD, not associated with *H. pylori* or NSAID use. The higher incidence might be attributable to molecular changes in gastric protective factors such as mucin among various races and ethnicities [18].

There existed a significant association between risk factors and PUD-related hospitalization. Patients with tobacco abuse were at 2.5 times higher odds of being hospitalized for PUD in our study, which is consistent with the theory of it being a potential environmental contributory factor in the development of PUD. Cigarette smoking is a proven risk factor for both duodenal and gastric ulcers. Smoking reduces epidermal growth factor and prostaglandin levels, impairing the gastric mucosal cell renewal process and increasing the risk of ulceration. It also weakens the angiogenesis process, reducing the ability to heal [19]. Cigarette smoking is also associated with a higher mortality rate among PUD patients [20].

Alcohol abuse had the highest rate of hospitalization secondary to PUD in our study. However, the causal association with PUD has not been established in the literature despite its known potential to cause acute gastric injury. The hypothesis is that alcohol could cause gastric ulcers by increasing gastric acid production through parietal cells. Additionally, it is not uncommon to find co-occurring tobacco use or cigarette smoking among alcohol abusers. The synergistic effect of alcohol and smoking could explain the increased prevalence of PUD [21]. Furthermore, PUD is more prevalent in cirrhotic patients with poor outcomes in this population, which coincides with our study results which found increased comorbid liver disease rate with PUD hospitalizations [22]. Portal hypertension caused by the cirrhotic liver is an important predictor for the development of PUD. Higher the venous pressure gradient, the greater the risk of ulcers [23].

Cannabinoids may be seen as prospective gastroprotective agents as they reduce gastric motility and acid secretion while relaxing the lower esophageal sphincters, potentially aiding in the healing of gastric ulcers [9,10]. However, the odds of association between CUD and hospitalization for PUD were statistically significant in our study. Among the inpatients with PUD, 3.2% had comorbid CUD. Cannabis has the potential to increase the risk of inflammation, which factors into causing ulcers. It also reduces pain sensitivity, which could mask ongoing inflammation and lead to increased incidence of ulcers. Past studies have established the detrimental effects of cannabis on GI health. A cross-sectional study found that CUD had 40.7% higher odds for irritable bowel syndrome (IBS) hospitalizations and worsened IBS and quality of life [24]. Another study investigating the relationship between CUD and persistent vomiting-related hospitalization found that CUD was independently associated with a 609% increased likelihood of persistent vomiting-related hospitalization [25]. Moreover, polysubstance abuse is common among cannabis users and could be a confounding factor in increasing the rates of PUD in this group. CUD was prevalent among males and blacks in our PUD inpatients, and a similar trend is supported by the current literature [26].

There were some limitations to our study. First, we used administrative data, so the inpatient data were included based on diagnostic codes, leading to underreporting of comorbidities and lack of patient-level clinical information due to which we did not have any information about whether the patients were on any ulcer preventive therapy like histamine h2 antagonists, proton pump inhibitors, or antacids. Further, despite a larger inpatient sample, the prevalence of PUD was very low. Second, as the identification of comorbid CUD was based on diagnostic codes, we could not know the amount and time of consumption of cannabis prior to the PUD hospitalization. Lastly, we used inpatient data that could not prove the causal relationship between the risk factors and PUD. The major strength of our study includes nationwide data analysis covering 44 states across the United States. Our results have external validity to the general population of the United States to assess the risk factor and the association of PUD. Information is coded independently by individual practitioners, so it is subject to minimal reporting bias.

## Conclusions

PUD was more prevalent among whites and males. CUD was a lesser prevalent comorbid substance use in PUD inpatients, yet it significantly increased the likelihood of PUD-related hospitalization by 18%. With the rising legalization of cannabis use, it is imperative to understand the impact of recreational cannabis use and/or CUD on a patient’s physical health and the related GI problems and to implement the screening for cannabis use and other substance use disorders for early diagnosis and management.
